# Intervenções para o Controle do Ritmo em Pacientes com Fibrilação Atrial – Anticoagulação Pré-Procedimento e Utilidade da Avaliação por Imagem do Átrio Esquerdo

**DOI:** 10.36660/abc.20220663

**Published:** 2022-10-05

**Authors:** Mirella Facin, Nelson Samesima

**Affiliations:** 1 Hospital das Clínicas Faculdade de Medicina Universidade de São Paulo São Paulo SP Brasil Instituto do Coração (InCor) – Hospital das Clínicas HCFMUSP – Faculdade de Medicina da Universidade de São Paulo , São Paulo , SP – Brasil

**Keywords:** Fibrilação Atrial, Cardioversão Elétrica, Ecocardiografia Transesofágica, Ecocardiografia Tridimensional, Trombose

A fibrilação atrial (FA) é arritmia sustentada mais comum na prática clínica, afetando 2-4% de todos os adultos no mundo. ^1 , 2^ Sua prevalência é ainda maior nos idosos e chega a quase 10% naqueles com mais de 80 anos. ^1^ De acordo com as estimativas atuais, um em cada três adultos com 55 anos desenvolverá FA durante a sua existência, com impacto substancial nas políticas de saúde e economia.2 As principais complicações da doença relacionam-se a eventos tromboembólicos (ET) e sintomas arrítmicos, ambos considerados alvos centrais no manejo de pacientes com FA. ^1 , 2^

A fibrilação atrial confere um risco 2-5 vezes maior de ET, que varia entre os pacientes acometidos de acordo com modificadores individuais. ^2^ Fatores de risco importantes resumidos no escore CHA _2_ DS _2_ -VASc – Insuficiência cardíaca congestiva, Hipertensão, Idade ≥ 75 anos, Diabetes, AVC/AIT, Doença vascular, Idade 65-74 anos, Sexo (feminino) – podem prever o risco de AVC, mitigado em quase 70% pela anticoagulação adequada. Os antagonistas da vitamina K (AVKs) foram os únicos anticoagulantes orais (ACOs) disponíveis por mais de meio século. De 2009 a 2013, os novos anticoagulantes orais ou anticoagulantes orais diretos (ACODs) foram apresentados à comunidade científica por quatro estudos randomizados controlados. ^3 - 6^ Esses medicamentos apresentaram eficácia semelhante aos AVKs na prevenção de eventos tromboembólicos, com melhor perfil de segurança contra sangramentos maiores – principalmente hemorragia intracraniana – e perfil farmacocinético e farmacodinâmico mais previsível, descartando a necessidade de monitoramento laboratorial de rotina. ^7^ No entanto, a aplicabilidade dos novos anticoagulantes orais em contextos diferentes daqueles testados, como na prevenção de AVC durante intervenções para controle do ritmo, foi tema de debate por alguns anos. A estratégia de controle do ritmo engloba tratamentos com o intuito de restaurar e manter o ritmo sinusal em pacientes com FA, tais como cardioversão, antiarrítmicos e ablação por cateter. ^2^ Essa abordagem é formalmente indicada para reduzir sintomas e melhorar a qualidade de vida após falha ou intolerância às drogas antiarrítmicas classe I ou III. ^2^ Atualmente, porém, há uma tendência à indicação precoce de procedimentos de controle do ritmo, na tentativa de evitar o remodelamento atrial e adiar a progressão da FA. ^1 , 2^ Entretanto, a cardioversão e a ablação por cateter podem precipitar eventos tromboembólicos em pacientes com FA, por deslocamento de trombos pré-existentes ou formação de novos trombos por diferentes mecanismos como o atordoamento atrial e a aderência à superfície trombogênica do equipamento de ablação ou ao endotélio fragilizado após a aplicação da radiofrequência. ^2 , 8^ Logo, a presença de trombos cardíacos contraindica os procedimentos de cardioversão e a ablação. ^9^ Na FA com duração superior a 48 horas, o risco tromboembólico periprocedimento pode chegar a 5-7% sem a profilaxia adequada. ^8 , 10^

A maioria dos tromboêmbolos relacionados à FA origina-se do apêndice atrial esquerdo. ^8 , 10^ No entanto, a prevalência de trombos no átrio esquerdo (AE) varia significativamente na literatura, de 0,6% a 27%, a depender das características da população e do tratamento utilizado. ^8 , 10^ O uso de AVKs, com tempo adequado na faixa terapêutica (INR 2,0-3,0) por pelo menos três semanas antes da restauração do ritmo sinusal, diminui consideravelmente as taxas de acidente vascular cerebral e tromboembolismo. ^2^ Sub-análises dos ensaios clínicos randomizados RE-LY, ROCKET-AF e ARISTOTLE demonstraram que os ACODs também foram bem-sucedidos nesse cenário. ^2 , 7^ As diretrizes atuais, portanto, recomendam anticoagulação oral terapêutica com AVKs/ACODs por ≥ 3 semanas antes de qualquer tentativa de controle do ritmo. ^1 , 2 , 11 - 13^ Se isso for inviável, por razões práticas ou de urgência, a avaliação do AE em busca de trombos através do ecocardiograma transesofágico (ETE) pode ser realizada. ^2 , 13^ Porém, o período de anticoagulação pré-procedimento sugerido nas diretrizes foi estabelecido de maneira arbitrária conforme o tempo presumido necessário para endotelização ou resolução de trombo de FA pré-existente. ^2^ Ademais, tais recomendações se basearam em estudos que examinaram complicações tromboembólicas periprocedimento. Dados sobre a prevalência de trombo atrial esquerdo em indivíduos que receberam anticoagulação de acordo com as diretrizes são escassos. ^9^ A maioria dos trabalhos observacionais com dados do mundo real possui claras limitações, como a falta de comparação simultânea entre os diferentes anticoagulantes orais e posologias e a inobservância de fatores de confusão, como período de uso de ACO e tempo na faixa terapêutica antes do ETE.

O trabalho de Marques et al., ^14^ na edição atual da ABC Cardiol, trouxe informações relevantes nesse campo. Os autores investigaram a presença de trombo atrial esquerdo e contraste denso espontâneo (CDE) em uma coorte retrospectiva unicêntrica que incluiu 354 pacientes submetidos a ETE antes da cardioversão por corrente contínua ou ablação por cateter de FA. Todos os pacientes receberam ≥ 3 semanas de ACODs (Dabigatrana 99, Rivaroxabana 222 e Apixabana 79). Nessa coorte, trombos no AE estavam presentes em 2,8% e contraste denso espontâneo em 7,3% dos pacientes. ^14^ Ambas as alterações ocorreram com maior frequência em indivíduos com idade mais avançada e escores CHA _2_ DS _2_ -VASc mais altos, e naqueles com átrio esquerdo aumentado e função ventricular esquerda reduzida. ^14^ Não houve diferença estatisticamente significante na taxa de trombos no AE e CDE entre os três anticoagulantes orais diretos testados. ^14^ Esses dados alinham-se com os achados de uma metanálise recente que incluiu 14.653 indivíduos e encontrou uma taxa não desprezível de 3% de trombo atrial esquerdo em pacientes anticoagulados com FA ou flutter atrial, com maior prevalência nos portadores de fibrilação atrial não-paroxística e escore CHA _2_ DS _2_ -VASc ≥ 3, independentemente do ACO utilizado. ^9 , 14^

Em essência, a anticoagulação oral contínua produz baixas taxas de AVC periprocedimento, que são semelhantes em todos os ACOs disponíveis. ^2^ No entanto, ao encontro do conhecimento já existente, Marques et al. demonstraram que, apesar da anticoagulação adequada, alguns pacientes podem apresentar trombo atrial esquerdo ou CDE, ^14^ fato que sugere a necessidade do uso mais criterioso do ETE, embasado no risco individual, com o objetivo de melhorar a segurança das intervenções de controle do ritmo em pacientes com FA.
