# Supplementary material for: Intervenções para o Controle do Ritmo em Pacientes com Fibrilação Atrial – Anticoagulação Pré-Procedimento e Utilidade da Avaliação por Imagem do Átrio Esquerdo
Source: Arq Bras Cardiol. 2022 Oct 5;119(4):520–1. [Article in Portuguese] doi: 10.36660/abc.20220663 (PMC9563887; doi:10.36660/abc.20220663)
Supplement: Translation

# Translation: Rhythm Control Interventions in Patients with Atrial Fibrillation – Insights on Preprocedural Anticoagulation and Utility of Left Atrial Imaging

**Authors:** Mirella Facin, Nelson Samesima

**Affiliations:** 1 Brazil Instituto do Coração (InCor) – Hospital das Clínicas HCFMUSP – Faculdade de Medicina da Universidade de São Paulo, São Paulo, SP – Brazil

**Keywords:** Atrial Fibrillation, Electric Countershock, Transesophageal Echocardiography, Thrombosis

The most common sustained arrhythmia in clinical practice is atrial fibrillation (AF),^1 , 2^ affecting 2-4% of the adult population worldwide. It is even more frequent with aging, with almost 10% prevalence in individuals older than 80.^1^ Current estimates state that one in every three adults aged 55 years will develop AF during their lifetime, leading to substantial healthcare and economic burden.^2^ Clinical issues relate primarily to thromboembolic events (TE) and arrhythmic symptoms, both central targets while managing patients with AF.^1 , 2^

Overall, atrial fibrillation confers a 2-5-fold escalated risk of TE, which is not evenly distributed, depending on unique modifiers.^2^ Important risk factors abridged in the CHA_2_DS_2_-VASc score – Congestive heart failure, Hypertension, Age ≥75 years, Diabetes, Stroke/TIA, Vascular disease, Age 65-74 years, Sex (female) – may predict stroke risk, consistently mitigated in almost 70% by proper anticoagulation. Vitamin K antagonists (VKAs) were the only oral anticoagulants available for over half a century. From 2009 to 2013, pivotal randomized controlled trials acquainted the scientific community with the new/direct oral anticoagulants (DOACs).^3 - 6^ These drugs not only held similar efficacy to VKA in preventing thromboembolic events but also had a better safety profile against major bleeding – notably intracranial hemorrhage – and a more predictable pharmacokinetic and pharmacodynamic profile, ruling out the need for routine laboratory monitoring.^7^ However, the applicability of DOACs in off-label backgrounds, including stroke prevention during rhythm control interventions, remained unclear for many years.

Encompassing treatments such as cardioversion, antiarrhythmics and catheter ablation, the rhythm control strategy comprises efforts to restore and maintain sinus rhythm in patients with AF.^2^ This approach has formal indications for reducing symptoms and improving quality of life after failure or intolerance to class I or III antiarrhythmic drugs.^2^ Nowadays, there is a trend toward an early indication of rhythm control procedures, trying to avoid atrial remodeling and postpone AF progression.^1 , 2^ AF cardioversion and catheter ablation may precipitate TE events by dislodgement of pre-existing thrombi or different *de novo* thrombus formation mechanisms, such as atrial stunning and adherence to the ablation’s equipment thrombogenic surface or ablation sites with endothelial disruption.^2 , 8^ Hence, the presence of cardiac *thrombi* contraindicates cardioversion and ablation procedures.^9^ In AF lasting more than 48 hours, the periprocedural thromboembolic risk may reach 5-7% without adequate prophylaxis.^8 , 10^

Most AF-related thromboemboli stem from the left atrial (LA) appendage.^8 , 10^ However, the reported prevalence of LA-thrombi varies significantly, from 0.6% to 27%, depending on population characteristics and treatment status.^8 , 10^ VKAs, within adequate time in the therapeutic range (INR 2.0-3.0) for at least three weeks before sinus rhythm restoration, effectively decrease the rates of stroke and thromboembolism.^2^ Sub-analyses of the randomized controlled trials RE-LY, ROCKET-AF and ARISTOTLE demonstrated that DOACs were also successful in this setting.^2 , 7^ Current guidelines, thus, recommend therapeutic oral anticoagulation with VKAs/DOACS for ≥3 weeks before any rhythm control attempt.^1 , 2 , 11 - 13^ If that is unfeasible, for urgency or practical reasons, pre-procedural screening for LA thrombus with transoesophageal echocardiography (TOE) may be performed.^2 , 13^ However, the preprocedural anticoagulation period suggested in the guidelines was arbitrarily based on the assumed time needed for endothelialization or resolution of pre-existing AF thrombus.^2^ Moreover, these endorsements relied on trials examining periprocedural thromboembolic complications. Existing literature on LA thrombi prevalence in individuals receiving guideline-directed anticoagulation is scarce.^9^ Most observational studies reporting data on real-world experience are limited, lacking comparison between the diverse OACs or different posology in the same study and observance for confounders, like proper anticoagulation (sufficient period and adequate time within therapeutic range) before TOE.

The work of Marques et al.^14^ in ABC’s current edition added relevant insights into this field. The authors investigated the presence of left atrial thrombi and dense spontaneous contrast (DSC) in a retrospective unicentric cohort that included 354 patients undergoing TOE before direct current cardioversion or AF catheter ablation. All patients received ≥3 weeks of DOACs (Dabigatran 99, Rivaroxaban 222, and Apixaban 79). In this cohort, LA thrombi were present in 2.8% and DSC in 7.3% of the patients.^14^ LA thrombi and DSC were more frequent in individuals with more advanced age and higher CHA_2_DS_2_-VASc scores, and those with left atrial enlargement and reduced left ventricular function.^14^ There was no statistically significant difference in LA thrombi, and DSC rates between the three tested DOACs.^14^ These reported data aligned with a recent meta-analysis including 14,653 individuals that found a non-negligible 3% prevalence of LA thrombus in anticoagulated patients with atrial fibrillation or atrial flutter, with increased odds for patients with non-paroxysmal atrial fibrillation and a CHA_2_DS_2_-VASc score ≥3, irrespective of the OAC used.^9 , 14^

In essence, continued oral anticoagulation yields low periprocedural stroke rates, which are similar to all available OACs.^2^ However, along with the existing knowledge, Marques *et al.* demonstrated that, despite adequate anticoagulation, some patients may still present LA thrombi and DSC,^14^ suggesting the need for more individualized and risk-based use of TOE to improve the safety of rhythm control interventions in patients with AF.
